# Toothsome Topics

**Published:** 1904-01-15

**Authors:** R. B. Tuller


### TOOTHSOME TOPICS.

BY R. B. TULLER, D.D.S.

J. Dubkin Chumpley, D.D.S.,<br>

Dentist.<br>

Fine Gold Fillings a specialty. Bird Center.

A professional card.

You've seen 'em like that, haven't you?

Fearing that D.D.S. may be misunderstood or misinterpreted, he makes it plain that he is a dentist.

He's no common, every-day, all-around dentist, but one who makes a specialty of fine gold fillings.

What he does to fill in the time between fine gold fillings he does not say.

The chances are that he now and then packs in a "silver" filling or files up and polishes a rubber plate.

But he wants it distinctly understood that he dosen't do that every day. J. Dubkin Chumpley, D.D.S., Dentist, Fine gold fillings a specialty, signifies, as he looks at it, a kind of upper-crust dentist.

If you want to see him score set him to work on fine gold fillings.

Great-large whoppers, too, that take hours to insert—with rubber dam *in situ* during the entire operation.

Chumpley may sweat some and his back may be breaking, but he's got endurance—got it,maybe, on the college foot-ball team.

Perchance he'll tell the patient that *this filling* is one of the most difficult a dentist is called upon to build.

Only specialists in gold fillings would locate anything so difficult. Common, ordinary dentists would never consider anything but just common, ordinary amalgam in such a place.

And the patient? How does she—most always she—enjoy it?

Methinks I see a bedraggled-looking female take a long sigh of relief as she impatiently helps J. Dub. remove the dam. Weary, worn, agonized, nerve-wrecked and half paralyzed, nearly in a state of collapse, she departs and thinks, "Heavens! have I got to go through all that again for the one over on the other side?"

Well, she don't have to if Chumpley would just go and get wise on a little inlay work.

There are Chumpleys and Chumpleys, and again Chumpleys plus.

We've all been Chumpleys to a large extent, though we are but common, every-day dentists and do not specialize fine gold work as though the other fellow did not make a *bete noir* of himself in the same old way.

Now, haven't you thought you'd like to do better for your patrons in some way, and especially if it could be done in a way not less remunerative and saving time and strain on yourself?

Then why cling so devotedly and persistently to pounding in gold by a long, tedious method, when there is a better way?

A better way? Sure, a better way and a much easier way—ten times easier for the patient and really nine and ninetenths times easier for the dentist, possibly, in a large majority of cases.

The trend of progressive thought and action of up-to-date dentists is to eliminate as much as possible the horrors of the dental chair, or reduce hours of nerve-racking strain and misery to minutes, and send patients away cheerful and not dreading the next engagement like a death sentence.

When you went to college how much more than one way did you learn to fill a tooth with gold?—that one way malleting in cohesive gold pellet by often taking hours and sometimes the better part of a day?

There are other very satisfactory methods calling for an intimate knowledge of non-cohesive and of crystal gold.

But malleting cohesive gold, by a slow, tedious process, at best, has stood for years, and now, the most approved method of filling teeth, and hence that is taught almost or quite to the exclusion of any other in most colleges; hence the specialist in fine gold fillings.

After some years of regular practice are you always satisfied that every gold filling you put in is a "peach?"—that is to say good?

Do you feel that most of them will do what they are mainly intended to do besides looking gaudy—preserve the teeth?

That you "can't teach an old dog new tricks" is an old saw, but no dentist ought to allow himself to be compared to an old dog.

If you are not wise, get wise as soon as you can. The high tide of cohesive gold work for large restorations of decayed and broken down teeth has been reached and passed.

I don't mean to say that the use of cohesive gold for a part of our work is out of date and perhaps never will be, but large and conspicuous restorations will be done in some easier and better way.

Many of you don't like the idea of getting out of the cohesive gold-and-mallet rut, but you've got to or be classed with Chumpleys, and some day in the trite but slangy saying, your name will be Dennis, alias Mud.

You must keep up with the procession. Why not get into the band-wagon and be not only in the procession but at the head? MAKE INLAYS.

Don't scowl! Don't howl! You'll have to come to it or drop behind.

Porcelain inlays? Yes, your anterior teeth. And your posterior ones, too, if you want to.

Say, you can set a porcelain inlay on the buccal surface, down under the edge of the gum, perchance, easier than you can put amalgam there.

Now, hold on! Don't get excited. I didn't say make and set the inlay, for it takes some little time to bake up an inlay.

But what I do contend is that you can prepare the cavity with much less cutting, saving your patient and yourself and when inlay is made it can be placed as easily as anything if made to go exactly to place when pressed in.

And if the cementing has been done properly it will stick and stay and preserve.

While you are doing the baking, sitting at easy posture in your laboratory and your patient is taking it easy reading or has gone out to return as you have agreed upon. When he or she comes back the setting is a very easy matter, quickly done, as quick and easy as you'd insert an amalgam filling if you wish.

Porcelain inlays require some delicate skill and a good deal of painstaking effort in most cases, not to mention artistic taste and ability in shading and contouring; and, yes, after all they *sometimes come out.*

Great Peter! out?

Aye, 'tis true; they do. But take a little retrospect along those fine gold fillings you put in. Did any ever come out? Did any ever fail to protect though they staid in for a few months? Did the work you did after you perchance became a specialist in fine gold fillings always preserve?

What? Do you mean to say some came out? And some failed? Well, yes, of course; but, gee whiz! an inlay, why, an inlay should never have a fault.

Well, they don't have many—if the cavity is made right—if the inlay is made right—if it is set right.

The average gold filling is not a good filling because it does not preserve, though it may stick in a long time.

The average inlay—one not the very finest fit possible and not exactly correct in shade or contour—well, if it stays in, preserves—and it will stay in if conditions have been made right, same as a gold filling will stay in if the conditions peculiar to such work have been made right.

When one has become as familiar with inlay work as he is with gold filling his work will stand with equal or even greater certainty.

Don't you remember your miserable failures in your first cohesive gold work and even after you became quite expert?

But the purpose of this topic this time is not to give special pointers about porcelain inlays, but rather to emphasize the value of inlays so logical and correct and artistic and durable when made correctly, and their especial value in reducing if not entirely eliminating some of the terrors of the dental chair.

All I have said applies as well to gold inlays except as regards color. The display of glittering, gaudy gold is becoming more and more objectionable to refined tastes, and therefore porcelain is above gold in the esthetic sense, but gold inlays will preserve and they can be made exceptionally strong for masticating service in posterior teeth.

Gold inlays have been made for a good many years and have done everything expected of them. The process of making them seems to some a little difficult and complicated, but any one who can make a good cohesive-gold filling or a gold crown ought to soon turn his hand to making an inlay.

I have a method, original with me, which I would like to tell you about, and once you are familiar with it by trying to make a few in teeth set in plaster you can save a lot of time and trouble in many cases. When you become expert you can save fully two-thirds or three-fourths the time it ordinarily takes to build up large cohesive-gold fillings.

The cavity must be prepared with no under cuts. If any they must be filled up with cement and walls left smooth and sloping slightly outward. The margins may be beveled, same as for gold fillings. (The contrary is the case with porcelain—margins must be square.) In other words, the cavity must be made so that an impression of it would draw freely without dragging or distorting the material used.

In this process I take the impression in gold.

I will describe the process of making an inlay by my method of a simple, large, bowl-shaped cavity in a lower molar for instance.

Supposing cavity to be all ready I take a third or one-half of a sheet of non-cohesive gold (cohesive doesn't work so easily), and fold it over and over on itself until I have a square about the size of the top of tooth. Laying it over the cavity, which may be moist, I force it to the bottom with a little ball of cotton, then expand it to the walls and fit it carefully over the margins and into all fissures or recesses described by surface within margin of cavity. Into this I pack a wad of crystal gold—enough to fill it comfortably, treating it as I would so much putty. Now let patient bite on it. Pack down or add more as may be indicated. Especial pains is to be made to fit with some firmness all around the margin carrying to the bottom of every pit and fissure it is supposed to occupy when finished.

To remove it from tooth, take hold of the overlapping gold foil with pliers and gently coax it out. Put it bottom up on a piece of glass, flow investment over it, just a thin layer. When that is hard turn top up and on a wire screen hold it over Bunsen flame, put on 18 or 20-K solder and draw it down through till filled full, which will easily show. After trimming off surplus around margin it may be ground and finished a good bit, if not altogether, before setting, care being taken to not injure margins. I find my cutting pliers hold it best while finishing. Now put in cavity and burnish down margins, and after scoring the back to give hold for cement it is ready to set.

Wash out cavity and dry thoroughly and keep dry and then mix cement and set. Wipe away surplus cement and force down hard. A hot burnisher will hurry the crystallization of cement. Edges should again be burnished down close. After a few moments, paraffine may be made to flow over the margins and the work is done.

The time taken to make this inlay is, in expert hands, not over fifteen or twenty minutes if every thing is ready and convenient to work quickly.

Caution is needed, if a blow-pipe flame is used in soldering or the foil and crystal gold may be melted. I use most always a low Bunsen flame. Flux should be cautiously used, too.

Now, I have made some of these crystal gold inlays by packing the cavity without a matrix except a strip of thin gold laid in first to lift it out by and have soldered it without investing at all, though it is best to. Cover all but top with a coating of whiting and water. This is a quicker way still for simple cavities, but it takes experience to know just how to handle it and not melt a thin edge or run solder where not wanted.—*American Journal.*

